# A randomized trial assessing the efficacy of Silymarin on endometrioma-related manifestations

**DOI:** 10.1038/s41598-022-22073-8

**Published:** 2022-10-20

**Authors:** Negin Mirzaei, Shahideh Jahanian Sadatmahalleh, Safoura Rouholamin, Malihe Nasiri

**Affiliations:** 1 Department of Reproductive Health and Midwifery, Faculty of Medical Sciences, Tarbiat Modares University, Jalal Al-Ahmad Highway, Nasr Bridge, Tehran, 14115-111 Iran; 2 Department of Obstetrics and Gynecology, Faculty of Medical Sciences, Isfahan University of Medical Sciences, Hezar-Jerib Ave., Isfahan, 81746 73461 Iran; 3 Department of Basic Sciences, Faculty of Nursing and Midwifery, Shahid Beheshti University of Medical Sciences, Tehran, Iran

**Keywords:** Endocrinology, Health care, Medical research, Diseases, Reproductive disorders

## Abstract

To study the effect of silymarin on the Interleukin-6 (IL-6) level, size of endometrioma lesion, pain, sexual function, and Quality of Life (QoL) in women diagnosed with endometriosis. This randomized, double-blind placebo-controlled clinical trial was performed on 70 women with endometriosis which was divided into two groups of intervention and control. The intervention was 140 mg silymarin (or matching placebo) administered twice daily for 12 weeks. The volume of endometrioma lesions, the level of IL-6 concentration in serum, pain, sexual function, and QoL were analyzed before and after the intervention. The means of endometrioma volume (P = 0.04), IL-6 (P = 0.002), and pain (P < 0.001) were reduced significantly in the silymarin group after intervention. However, the QoL and female sexual function did not improve substantially in the two groups (P > 0.05). Silymarin significantly reduced interleukin-6 levels, sizes of endometrioma lesions, and pain-related symptoms. The trial has been registered in the Iranian Registry of Clinical Trials (IRCT20150905023897N5) on 4th February 2020 (04/02/2020) (https://en.irct.ir/trial/42215) and the date of initial participant enrollment was 2nd March 2020 (02/03/2020).

## Introduction

Endometriosis is a debilitating recurrent gynecological disorder characterized by the proliferation of the endometrial tissue in ectopic sites which is most commonly found in the ovaries, affecting approximately 10% of reproductive-aged women^1,2^. Ovarian endometriosis (OMA) might be accompanied by several pain-related manifestations such as pain, dysmenorrhea, dyspareunia, and subfertility^3^. Not only do these discomfort symptoms pose devastating impacts on the Quality of Life (QoL), psychological, social, and sexual life of afflicted women, also these can represent a massive economic burden on patients and the health care system^4,5^.

Although the accurate pathogenesis of endometriosis remains elusive, several therapeutic modalities for endometriosis are suggested based on its unclear mechanisms. Therefore, Oral Contraceptive Pills (OCPs), progestogens, anti-progestogens, Gonadotrophin-Releasing Hormone analogues (GnRH-a), and surgical therapy, alone or in combination are used for treatment^6^. Recent studies have addressed the roles of Oxidative Stress (OS) and inflammatory factors in the etiology and improvement of OMA^7^. Hence, an endometrioma contains a higher level of free iron, Reactive Oxygen Species (ROS), and inflammatory molecules such as Interleukin (IL)-8, IL-6, Tumor Necrosis Factor-a (TNF-a) compared with those present in peripheral blood or other types of benign cysts^8,9^. Thus, endometriosis treatment should not be different from that of other inflammatory disorders^10^.

Silymarin is a unique flavonoid complex derived from the milk thistle plant (Silybum marianum) and consists of a family of flavolignans such as silybins A and B **(**major bioactive compounds), silychristin, and silydianin^11^. Silymarin displays potent antioxidant and free radical scavenging abilities and has strong anti-fibrotic properties^12,13^. The antioxidant mechanism of silymarin results from inducing superoxide dismutase, increasing cellular glutathione content and inhibiting lipid peroxidation, it also is a powerful iron chelator^14^. Apart from antioxidant effects, silymarin acts an anti-inflammatory agent by inhibiting the migration of neutrophils to the site of inflammation, Kupffer cells, prostaglandins, leukotrienes, and transcription factor NF- κB which regulates various genes involved in the inflammatory process^15–17^. It is also has been reported that silymarin can inhibit the TNF-a, interferon-c, IL-2, and Inducible Nitric Oxide Synthase (iNOS)^14^. In addition, several in vitro and animal studies have verified the effects of silymarin in decreasing the size and histopathologic grade of the endometrial lesions^18–20^.

Thus, in light of previous studies on silymarin in other chronic disease and endometrial cells, we explored the relationship between silymarin and OMA in a double-blind, randomized clinical trial looking at the effect of silymarin on cessation of pain-related symptoms in women suffering from OMA.

## Materials and methods

### Trial design and overview

This study was a phase II, double-blind, randomized, placebo-controlled trial conducted in gynecological clinics affiliated with Tehran University of Medical Sciences, Tehran, Iran, from 2nd March 2020 (02/03/2020) to 18th May 2021 (18/05/2021). This clinical trial comprised a 12-week treatment period, after which some of the endometriosis-related manifestations were evaluated. This trial conformed to the ethical guidelines of the 1975 Declaration of Helsinki, and ethical approval was obtained from the Ethics Committee of Tarbiat Modares University, Tehran, Iran, before the commencement of the study on 22nd October 2019 (IR.MODARES.REC.1398.143). In addition to that, it has been registered in the Iranian Registry of Clinical Trials in 4th February 2020 (04/02/2020) (IRCT20150905023897N5). All potential study subjects provided written informed consent and were informed about the aims and possible risks of the trial before the study. Not only can all authors attest to the completeness and accuracy of the data and analyses and adherence to the trial protocol, but also they have reviewed and approved the final manuscript.

### Participants

Eligible subjects were women of reproductive age (15–49 years), married, and non-pregnant with a confirmed diagnosis of OMA via transvaginal ultrasonography performed by a single sonologist at the three-dimensional level in the previous five years and currently experiencing moderate to severe pain and receiving 2 mg dienogest daily in last six months [Dienogest as a conservative treatment option manages endometriosis through down-regulating estrogen receptors and constraining the local production of estradiol^21^].

The pattern recognition using subjective evaluation of Gray-scale and Doppler-ultrasound characteristics based on IOTA rules “an adnexal mass with ground glass echogenicity of the cyst fluid, one to four locules and no papillations with detectable blood flow” and volume (L × D × W × 0.5235) was achieved through the Orsini formula^22^.

Women would have been excluded if they had suffered from chronic diseases such as hypertension, diabetes, coronary, renal, and hepatic disorders or liver enzyme anomalies. Also, suppose they had taken specific medications like oral contraceptives, anti-depressant and GnRH analogues, or systemic glucocorticoids, with a specified wash-out period for each one based on the period. In that case, they might have a continued effect. In addition, using silymarin or other milk thistle preparations and anti-inflammatory supplements (e.g., vitamin, vitamin E,…) and even non-prescribed complementary alternative drugs must have been stopped for at least 30 days before the study. All subjects were instructed to use non-hormonal, double-barrier contraception such as a condom or diaphragm, each combined with a spermicide.

### Interventions

Eligible subjects were randomized into placebo and treatment groups based on the computer-generated list. All participants received a dose of 280 mg silymarin including two tablets of Livergol 140 mg, Goldaru Pharma Co. Isfahan-Iran) or placebo (Goldaru Pharma Co. Isfahan-Iran) daily in two meals (after breakfast and dinner) for 12 weeks along with standard treatment of endometrioma (dienogest 2 mg/day). There is evidence that medical treatment, in particular with progestogens, is effective in reducing pain symptoms and size of endometrioma lesions^23^. In the category of progestogens, dienogest is a fourth-generation selective progestin with a substantial local effect on endometriotic lesions in women have been taking dienogest 2 mg daily for at least six months^24,25^. Therefore, all of the participants had taken dienogest for at least 6 months. This dosage was chosen because it is safe and effective in treating liver diseases^26^. The therapeutic doses of silymarin have been considered safe and well-tolerated in humans and it has low risks of drug interactions^27^. Some slight gastrointestinal discomforts like nausea and diarrhea might be accrued^28^, however, participants did not report any side effects related to silymarin.

### Randomization and blinding

Simple randomization was done according to a computer-generated list of random number groups prepared using Statistical Analysis System Software Version 9.2 (SAS Institute Inc., Cary, NC, USA). All participants were randomly allocated to each arm and given the tablets based on a computer-generated randomization list by the investigator. Treatment and placebo allocations were blinded to subjects, the investigator, and the attending doctor.

### Trial procedures and assessments

At the first and follow-up visit that was planned after 12-week treatment, participants were referred to the lab and sonography single-center (based on routine standard protocols) to measure the concentration of IL-6 in serum by Enzyme-Linked Immunosorbent Assay (ELISA) using ELISA kit provided by Siemens (Berlin and Munich, Germany, pg/mL) and the sensitivity of the IL 6 assay was 0.038 pg/mL and inter and intra-assay coefficients of variation were less than 9.8 and 6.6%, respectively. Recording of the sizes of the endometrioma lesions via three-dimensional transvaginal ultrasonography (by the same physician in blind), respectively. In addition, they were asked to fill out several self-report questionnaires as follows:

Firstly, demographic data that consists of address, phone number, date of birth, weight, height, Body Mass Index (BMI), education level, occupation, education level of husband, and a history of the disease was gathered.

Because the observation suggests that IL-6 may be a good marker of disease prediction and progression and may serve as biomarkers for the diagnosis of endometriosis^29–31^.

#### Female sexual function (FSFI)

FSFI investigates the sexual function and consists of 19 Likert-scale questions in six domains of desire, arousal, lubrication, satisfaction, orgasm, and pain. The lower score is, the more sexual function impaired^32^. The Iranian version of FSFI has been evaluated for both reliability and validity^33^.

#### Short form health survey (SF-12)

SF-12 is designed to evaluate the physical and mental components of health-related QoL by 12 questions. Lower scores indicate inferior health-related QoL^34^. The validity and reliability of this questionnaire have been confirmed in Iran^35^.

#### Visual analog scale (VAS)

VAS has been used most often to assess the intensity of endometriosis-related pain (dysmenorrhea, deep dyspareunia, chronic pelvic pain, and menstrual and non-menstrual dysphasia). It is an 11-point scale in which 0 represented the absence of pain, while 10 meant unbearable pain^36^.

### Outcome

One of the primary endpoints was a significant reduction in the mean pelvic pain assessed after 12-week treatment on a VAS. Other primary efficacy endpoints at weeks 12 included a significant decrease in the volume of endometrioma lesions diagnosed via transvaginal ultra-sonography and any change from baseline in the level of IL-6.

Secondary efficacy endpoints were improvement of individuals’ QoL and sexual function assessed on mentioned questionnaires.

### Statistical analysis

Based on the study results of Almassinokiani et al.^37^, who assessed the effects of vitamin D on endometriosis-associated pain, with a 99% confidence interval and 90% power of the test, the sample size was determined as 35 individuals for each group. By counting 20% sample loss, the minimum sample size was estimated at 40 individuals in each group, meaning that 80 women diagnosed with endometrioma who had the inclusion criteria (40 persons in each group) were recruited for the present study.

$${\text{n}} \ge 2 \frac{{({\mathbf{z}}_{{\varvec{\upalpha}}/2}+{\mathbf{z}}_{{\varvec{\upbeta}}})}^{2}{{\varvec{\upsigma}}}^{2}}{{({{\varvec{\upmu}}}_{1}-{{\varvec{\upmu}}}_{2})}^{2}}$$

where, α is the probability of type I errors (α = 0.05), and βindicates the probability of type II errors (β = 0.20). In addition, (${{\varvec{\upmu}}}_{1}$ − ${{\varvec{\upmu}}}_{2}$)/σ represents the size effect observed that is equal 0.7.

$$\mathbf{n}=2{\left(1.96+0.85\right)}^{2}{\left(\frac{1}{0.65}\right)}^{2}=33$$

All statistical analyses were performed by the SPSS software (ver. 22.0) (SPSS Inc., Chicago, IL, USA). The data of normality in distribution were examined by one sample K-S. Group comparisons were carried out with Student’s t-test and Chi-square test, and Paired T-test. P < 0.05 was considered statistically significant.

### Ethics approval and consent to participate

The study protocol was approved by the Ethics Committee of Tarbiat Modares university (code: IR.MODARES.REC.1398.143). All procedures were in accordance with the ethical standards of the Regional Research Committee and with the Declaration of Helsinki 1964 and its later amendments. After explaining the study's purposes, informed written consent and verbal assent were obtained from all participants. They were informed that their participation was voluntary, confidential and anonymous, and that they had the right to withdraw from the research at any time.

## Results

**Figure 1:**
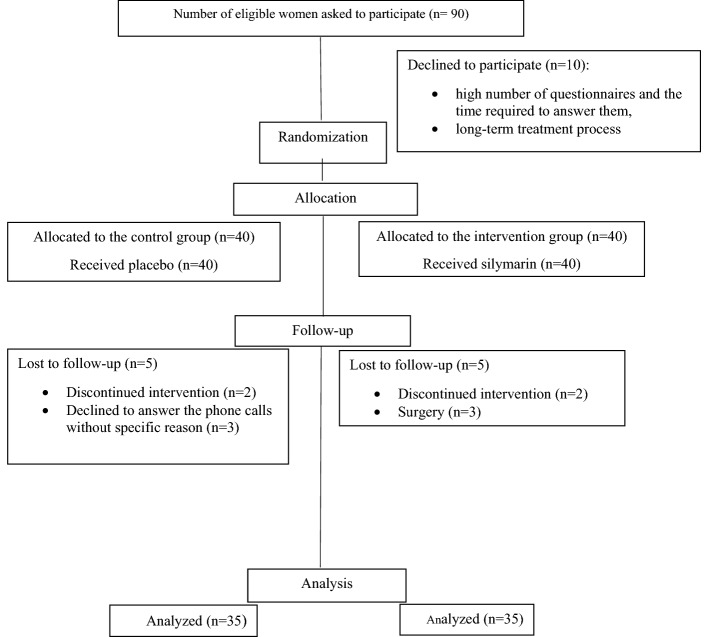
Flowchart of participants: women screened for eligibility, admitted to and randomized in the study on the use of the silymarin .

Overall, 10 out of the 90 potentially eligible women refused to participate in the study because of time consuming completing of questionnaires and longtime treatment process, so the response rate was approximately 88%. A total of 80 individuals, (40 women in each group) were enrolled into the study, 10 were excluded due to the unwillingness to peruse recommended treatment (n = 8), surgery (n = 3), the rate of sample loss was 12% (Fig. 1). Finally, 35 women were enrolled in each group.

Patients reported a mean of 3.4 years since the first diagnosis of endometriosis. Table 1 describes the characteristics of women in the intervention and control groups (the minimum age of participants was 21 years old). There was no statistically significant difference in women’s age, Body Mass Index (BMI), parity, educational level, and occupation status between the two groups (P > 0.05).

**Table 1:** Comparison of demographic characteristics between intervention and control groups.

Variable	Intervention group n = 35	Control group n = 35	P-value
Age of female*	36.49 ± 8.11	33.94 ± 7.00	0.16
Number of children**			
0	11 (31.4)	18 (51.4)	0.29
1	13 (37.1)	9 (25.7)	
2	10 (28.6)	6 (17.1)	
3 and more	1 (2.9)	2 (5.7)	
Education of female**			
Undergraduate	17 (48.6)	14 (40.0)	0.47
Postgraduate	18 (51.4)	21 (60.0)	
Occupation**			
Housewife	19 (54.3)	22 (62.9)	0.46
Employee	16 (45.7)	13 (37.1)	
BMI (kg/ m^2^) *	24.25 ± 4.02	24.54 ± 4.02	0.75

*BMI* Body Mass Index.

*Values are given as mean ± SD using t-test.

**Values are given as a number (%) using Chi-squared test.

As can be seen in Table 2, evaluation of the two groups with regard to FSFI before and after treatment did not show any significant differences. The total score of FSFI was significantly lower in control group after treatment (25.54 ± 2.74 vs 24.64 ± 2.74, P = 0.03).

**Table 2:** Comparison of FSFI scores between intervention and control groups.

Variable	Intervention group n = 35 Mean ± SD	Control group n = 35 Mean ± SD	P-value*
**Desire**			
Pre-assessment	3.58 ± 0.71	3.45 ± 0.66	0.42
Post-assessment	3.90 ± 0.75	3.50 ± 0.82	0.06
P-value**	0.01	0.72	
**Arousal**			
Pre-assessment	3.86 ± 1.18	4.01 ± 0.78	0.56
Post-assessment	4.21 ± 1.01	3.85 ± 0.78	0.15
P-value**	0.40	0.23	
**Lubrication**			
Pre-assessment	4.37 ± 1.22	4.87 ± 0.97	0.87
Post-assessment	4.53 ± 1.04	4.69 ± 0.83	0.54
P-value**	0.44	0.29	
**Orgasm**			
Pre-assessment	4.43 ± 1.25	4.68 ± 0.81	0.37
Post-assessment	4.45 ± 1.25	4.15 ± 0.91	0.31
P-value**	0.40	0.01	
**Satisfaction**			
Pre-assessment	4.50 ± 1.23	4.61 ± 0.88	0.90
Post-assessment	4.40 ± 1.04	4.36 ± 0.90	0.69
P-value**	0.54	0.06	
**Pain**			
Pre-assessment	3.73 ± 1.23	3.94 ± 1.35	0.44
Post-assessment	4.07 ± 0.76	4.05 ± 0.79	0.97
P-value**	0.18	0.50	
**Total score**			
Pre-assessment	24.39 ± 4.66	25.54 ± 2.74	0.25
Post-assessment	25.76 ± 4.33	24.64 ± 2.74	0.27
P-value**	0.28	0.03	

*FSFI* Female Sexual Function Index.

*T-test was carried out for comparison of the groups.

**Paired T-test was carried out for comparison of changes between the groups.

The comparison of SF-12 scores between the intervention and control groups is shown in Table 3. The mean total scores of SF-12 were not significantly different between groups before treatment compared with after intervention (P > 0.05).

**Table 3:** Comparison of the scores of subgroups of QoL between intervention and control groups.

Variable	Intervention group n = 35 Mean ± SD	Control group n = 35 Mean ± SD	P-value*
**Sum score of physical components (PCS-12)**			
Pre-assessment	76.23 ± 13.59	72.03 ± 18.95	0.31
Post-assessment	78.25 ± 11.51	71.45 ± 20.52	0.13
P-value**	0.91	0.71	
**Sum score of mental components (MCS-12)**			
Pre-assessment	69.79 ± 14.50	70.72 ± 13.80	0.79
Post-assessment	73.20 ± 14.33	72.43 ± 12.20	0.83
P-value**	0.47	0.36	
**Total score**			
Pre-assessment	73.01 ± 11.73	71.51 ± 14.34	0.65
Post-assessment	75.73 ± 11.56	72.19 ± 15.35	0.34
P-value**	0.58	0.37	

*QoL* Quality of Life.

*T-test was carried out for comparison of the groups.

**Paired T-test was carried out for comparison of changes between the group.

It can be seen from the data in Tables 4 and 5 that there was significant difference in intervention group for the pelvic pain mean changes from baseline to treatment end (7.29 ± 2.49 vs 4.88 ± 2.00, P < 0.001). In addition, IL-6 levels decreased significantly during the 90-day study period (2.40 ± 0.52 vs 2.12 ± 0.20, P = 0.002).

**Table 4:** Comparison of the serum levels of IL-6 (pg/mL) between intervention and control groups.

**Variable**	Intervention group n = 35 Mean ± SD	Control group n = 35 Mean ± SD	P-value*
**IL-6**			
Pre-assessment	2.40 ± 0.52	2.57 ± 0.78	0.29
Post-assessment	2.12 ± 0.20	2.42 ± 0.73	0.03
P-value**	0.002	0.30	

IL-6: Interleukin 6, pg/mL (picograms per milliliter).

*T-test was carried out for comparison of the groups.

**Paired T-test was carried out for comparison of changes between the groups.

**Table 5:** Comparison of the Visual Analog Pain Scale between intervention and control groups.

Variable	Intervention group n = 35 Mean ± SD	Control group n = 35 Mean ± SD	P-value*
**Pelvic pain (VAS)**			
Pre-assessment	7.29 ± 2.49	6.73 ± 2.97	0.41
Post-assessment	4.88 ± 2.00	6.61 ± 2.64	0.01
P-value**	< 0.001	0.64	

*T-test was carried out for comparison of the groups.

**Paired T-test was carried out for comparison of changes between the groups.

Table 6 shows a comparison between the 2 groups for the endometrioma lesions volumes at before and after treatment. There were significant differences between the volumes of endometrioma lesions in right ovary intervention group.

**Table 6:** Comparison of the ovaries’ volume between intervention and control groups.

Variable	Intervention group n = 35 Mean ± SD	Control group n = 35 Mean ± SD	P-value*
**Right ovary**			
Pre-assessment	58,863.51 ± 83,820.56	75,848.03 ± 135,023.70	0.59
Post-assessment	33,694.85 ± 53,124.60	81,963.81 ± 150,300.15	0.24
P-value**	0.04	0.16	
**Left ovary**			
Pre-assessment	65,773.67 ± 76,725.35	54,857.73 ± 57,983.99	0.60
Post-assessment	58,079.16 ± 73,622.66	70,801.58 ± 93,146.68	0.66
P-value**	0.23	0.26	

*T-test was carried out for comparison of the groups.

**Paired T-test was carried out for comparison of changes between the groups.

## Discussion

To the best of our knowledge, this is the first double-blind, randomized clinical trial conducted to investigate the effect of silymarin on serum IL‐6 Levels, the size of endometrioma lesions, QoL, pain, and sexual function in women diagnosed with OMA.

This study confirms that silymarin can be effective in reducing the size of endometrioma lesions is consistent with previous observations in animal models. Jouhari et al. compared the effects of silymarin, Cabergoline, and Letrozole on induced endometrial lesions in rats for about 6 months. Silymarin, Letrozole, and Cabergoline administration could decrease size and histopathologic grade of the induced endometrial lesions significantly and those who were received silymarin had significantly higher serum total antioxidant activity compared to control after 21 days^18^.

Nahari et al. evaluating legions establishment and growth in experimentally-induced endometriosis in 12 rats after 28 days of oral administration of silymarin showed that the combination of enhancing ERK1/2 expression and suppressing the Bcl-2 expression by silymarin accompanying with down-regulating the angiogenesis ratio led to promote the apoptosis pathway, consequently induced severe fibrosis in endometriotic-like legions^19^.

A recent prospective study showed that not only could silibinin induce OS and lipid peroxidation in human endometriotic cells, but also it exerted antiproliferative and apoptotic impacts on human endometriotic cell lines VK2/E6E7 and End1/E6E7^20^. Moreover, the effects of silibinin on reducing endometriotic lesions by inhibiting the expression of inflammatory cytokines was verified by using an animal model mimicking the retrograde menstruation hypothesis in mice and they proved the possibility of effectiveness of silibinin as a novel therapeutic agent or supplement in inhibiting progression of endometriosis in vitro and in vivo^20^.

The results also showed that oral administration of silymarin 280 mg/day decreased the IL-6 concentration levels significantly during the study period within the intervention group. Arafa Keshk et al. evaluating the potential protective effects of silymarin against indomethacin-induced gastric injury in rats, have demonstrated that suppression in gastric inflammation by decreasing myeloperoxidase activity, TNF- α, and IL-6 expression levels along with NF-κB expression. Meanwhile, silymarin prevent gastric OS via inhibition of lipid peroxides formation, enhancement of glutathione peroxidase, superoxide dismutase activities and up-regulation of Nuclear Factor-Erythroid-2-Related Factor 2 (Nrf2), the redox-sensitive master regulator of OS signaling^38^.

Additionally, Wei Zhang et al. showed that silymarin could protect against the liver injury caused by ethanol administration as it markedly downregulated the expression of NF-κB p65, ICAM-1 and IL-6 in liver tissue^39^.

Rongjuan Zheng et al. evaluated the chemo-preventive effect of silibinin (major component of silymarin) on a Colitis-Associated Cancer (CAC) mouse model and determined its impact on IL-6/STAT3 signaling. Silibinin decreased the amount and size of tumors in mice accompanying with inhibition of colonic tumor cell proliferation and promotion of cellular apoptosis. Furthermore, they showed that silibinin could reduce the production of inflammatory cytokines and can protect against colitis-associated tumorigenesis in mice via inhibiting IL-6/STAT3^40^.

In addition, it is revealed that silymarin attenuate the expression of NF-kB and the subsequent inflammatory cascade by suppressing IκB, effectively can down-regulate the expressions of TPA-induced IL-1β, IL-6, TNF-α, and COX-2 in a dose-dependent manner^41,42^.

Numerous mechanisms are involved in endometriosis-related pain such as various algogens (pain-producing agents), cytokines (such as IL-1b, IL-6, and TNF-a), growth factors (such as b-nerve growth factor and vascular endothelial growth factor), and several chemokines, such as CCL2^43,44^.

Several papers have investigated that different kind of antioxidant such as vitamin E and vitamin C can reduce peritoneal inflammatory markers and decrease chronic pelvic pain in endometriosis women^45–47^. It might be speculated silymarin could be effective in reducing pain as an antioxidant agent which is at least ten times more potent than vitamin E can suppress nitric oxide, prostaglandin E2 (PGE2), leukotrienes, cytokines production, and neutrophils infiltration^48–50^. This study supports evidence from previous observations about the analgesic effects of silymarin.

The exact physiopathology of endometriosis is poorly understood. Inflammation, however, is known as a main factor in initiation and progression of endometriosis. Hence, inflammatory mediators such as IL‐1β, IL‐6, IL‐8 increase in the peritoneal, serum, and endometrium of endometriosis patients, leading to enhancing proliferation and decreasing apoptotic rate in endometriotic cells^51,52^. In addition, the expression of NF-κB, Cyclooxygenase‐2 (COX‐2), Vascular Endothelial Growth Factor (VEGF), IL‐6, TNF‐α, IL‐8, C‐C Motif Chemokine 2 (MCP‐1), and IL‐10 have increased in endometriosis cases^53,54^. An inflammatory response is induced by cytokines and prostaglandins, which produce inflammation at the site of endometriotic implantation^55^. Following this inflammatory response, new vascularization and new fibrous tissue formation are initiated^55^. This causes adhesions and pain associated with this disease process as a result of the snowball effect. Moreover, these issues also contribute to the most common complications of this disease, such as infertility and chronic pelvic pain. In comparison with patients with stage one or two endometriosis, patients with endometriomas usually suffer from a more severe disease state and experience this to a greater extent^56^.

The anti-inflammatory effects of silymarin is mediated through the inhibition of NF-κB regulated gene products including COX-2 and Prostaglandin E2 (PGE2), as well as inflammatory cytokines^57^, through suppressing the degradation of Inhibitory kappa B (IκB) degradation, preventing both the translocation of NF-κB into the nucleus and the activation of gene transcription associated with inflammation^58^.

Oxidative Stress, an imbalance between ROS and antioxidants, is known as a main factor in the endometriosis pathogenesis^59^. Also, increased peritoneal oxidized low-density lipoprotein levels and ROS production by macrophages are observed in association with an altered expression of endometrial antioxidant and pro-oxidant enzymes^59^. In addition, Compared to healthy women, endometriotic cells increase the expression of glutathione peroxidase and Superoxide Dismutase (SOD) activity, while catalase concentrations are lower^60^. By eliciting c-Fos and c-Jun expression, OS activates the Mitogen-Activated Protein Kinase (MAPK)/Extracellular Signal-Regulated Kinase (ERK) pathway and promotes the survival and proliferation of endometriotic lesions. Proliferative responses are influenced by the ERK signaling pathway through the production of endogenous ROS^61^. Moreover, the activity of antioxidant system is less in endometriosis patients than healthy subjects^62^. Endometriosis subjects exhibit elevated growth, proinflammatory, and angiogenic mediators, as a result of ROS generation produced in peritoneal macrophages by iron overload^63^.

Silymarin is considered as an antioxidant agent via several mechanisms that include scavenging free radicals and chelating metals-promoters such as Fe and Cu, preventing ROS formation enzymes e.g. NAPDH Oxidases, activates antioxidant enzymes and inhibiting of lipid peroxidation, regulating the cell membrane permeability and increasing the stability, increases ribosomal protein synthesis by stimulation RNA polymerase, and regulation of signaling through activation of Nrf2 and inhibition of NF-κB^64^.

According to the fact that endometriosis is a very complex condition with wide range of clinical features including pelvic pain, dyspareunia, infertility, and menstrual irregularities that might compromise QoL, psychological wellbeing, sexual function, and interpersonal relationships of affected women^65,66^, Caruso et al. evaluated the effects of dienogest on QoL and sexual function in endometriosis women, reported that progressive reduction of the pain could contribute in improving the QoL and sexual life^67^.

Very little was found in the literature on the question of the effectiveness of silymarin on QoL and sexual function. Gillessen et al. investigating the effect of silymarin on liver function and QoL in a non-interventional study, reported that improvement in liver-related symptoms and increased QoL after 2 months^68^.

Previous researches had proved that silymarin and other anti-oxidants agents can enhance QoL in patients because of their positive effects on pain reduction^69,70^. However, the present study found inconsistent results and there was neither a significant difference in SF-12 between treatment and placebo groups nor within groups pre/post treatment.

The strengths of the study are the RCT design and novelty. In addition, as maintaining endometriosis associated women on a placebo is unethical, the intervention was recommended along with routine treatment. However, the principal limitation of the study is the 8% loss to follow-up and the short duration of follow-up; therefore, results probably should be extrapolated by long-term follow up. Additionally, these data warrant further investigations for the potential use of silymarin in the management of endometriosis features to assess long-term effects of this treatment by larger sample. One of the major limitations of this study is poor oral bioavailability of silymarin due to its poor enteral absorption, instability in the gastric environment, and poor solubility. Thus, silymarin must be administered at a higher dose in order to enhance therapeutic efficacy^71^. According to the promising effects of silymarin on chronic diseases, the use of nanotechnological strategies appears can improve its bioavailability and provide a prolonged silymarin release at the site of absorption^72^.

## Conclusion

In conclusion, silymarin can be considered as a feasible option for treatment women with endometrioma-associated symptoms that has few side effects.

## Data Availability

The data sets used and analyzed for the current study are available upon reasonable request of the corresponding author Dr. Shahideh Jahanian (shahideh.jahanian@modares.ac.ir).

## Abbreviations

IL: Interleukin
QoL: Quality of Life
COX‐2: Cyclooxygenase‐2
MCP‐1: C‐C Motif Chemokine 2 (MCP‐1)
PGE2: Prostaglandin E2
IκB: Inhibitory kappa B
ERK: Extracellular signal-regulated kinase
SOD: Superoxide dismutase
MAPK: Mitogen-activated protein kinase
VEGF: Vascular endothelial growth factor
TNF‐α: Tumor necrosis factor‐α
MCP‐2: C–C motif chemokine 2
OMA: Ovarian endometrioma
ROS: Reactive oxygen species
OCPs: Oral contraceptive pills
GnRH-a: Releasing hormone analogues
NF-κB: Nuclear factor kappa B p65
Nrf2: Nuclear factor-erythroid-2-related factor 2
CAC: Colitis-associated cancer
iNOS: Inducible nitric oxide synthase
pg/mL: Picograms per milliliter
FSFI: Female sexual function
SF-12: Short form health survey
VAS: Visual analog scale
ELISA: Enzyme-linked immunosorbent assay
